# The nucleocytoplasmic translocation and up-regulation of ING5 protein in breast cancer: a potential target for gene therapy

**DOI:** 10.18632/oncotarget.17918

**Published:** 2017-05-17

**Authors:** Xiao-Qing Ding, Shuang Zhao, Lei Yang, Xin Zhao, Gui-Feng Zhao, Shu-Peng Zhao, Zhi-Jie Li, Hua-Chuan Zheng

**Affiliations:** ^1^ Department of Experimental Oncology and Animal Center, Shengjing Hospital of China Medical University, Shenyang 110004, China; ^2^ Department of Surgery, The First Affiliated Hospital of Jinzhou Medical University, Jinzhou 121001, China

**Keywords:** breast cancer, ING5, pathogenesis, aggressiveness, progression

## Abstract

Here, we found that ING5 overexpression resulted in a lower proliferation, reduced glucose metabolism, S arrest, decreased migration and invasion, apoptotic induction, fat accumulation, autophagy, senescence and mesenchymal-epithelial–transition of breast cancer cells. It also suppressed the tumor growth of breast cancer cells by inhibiting proliferation, inducing apoptosis and autophagy. ING5-mediated chemoresistance was positively linked to Akt and NF-κB activation, MRP1 and GST-π overexpression, and FBXW7 hypoexpression. *ING5* expression was higher in breast cancer than normal tissue at both mRNA and protein levels. *ING5* mRNA expression was positively correlated with relapse- and distant metastasis-free survival rates. Nuclear ING5 expression showed gradual decrease from breast normal tissue, fibroadenoma, adenomatosis, primary to metastatic cancers, while versa for cytoplasmic ING5. Nuclear ING5 expression was negatively correlated with distant metastasis and p53 hypoexpression, while cytoplasmic ING5 expression was positively correlated with tumor size and ER expression. These data suggested that up-regulated expression and nucleocytoplasmic translocation of ING5 protein were observed in breast cancer. The higher expression of nuclear ING5 was inversely linked to worse clinicopathological behaviors of breast cancer by *in vivo* and vitro reversing aggressive phenotypes. Therefore, it should be employed as a biomarker to indicate the tumorigenesis and aggressiveness of breast cancer, and as a potential target for gene therapy.

## INTRODUCTION

Breast cancer is a leading cancer in women worldwide, and more common in developed countries. Its risk factors include female, obesity, lack of physical exercise, alcohol drinking, hormone exposure, ionizing radiation, early age at first menstruation, boring children late or not, older age, family history, and genetic alteration [1, 2]. Presently, it is more important to clarify the molecular mechanisms of breast cancer, and find out a potential target for early diagnosis and gene therapy.

Inhibitor of growth 5 (ING5) belongs to Class II tumor suppressor and structurally contains LZL (leucine zipper like), NCR (novel conserved region), NLS (nuclear localization signal), and PHD (plant homeo domain) from amino- to carboxyl-terminal. ING5 was reported to promote DNA repair, induce apoptosis and chromatin remodeling by forming histone acetyl transferase (HAT) complexes [3–5]. Additionally, it might activate the cyclin-dependent kinase inhibitor p21/waf1 promoter to induce p21 expression, enhance p53 acetylation at Lys-382 and Lys-120, and physically interact with p300 and p53 [6]. ING5 could also decrease cellular proliferation, and induce apoptosis in a p53-dependent manner [6]. Recently, its down-regulation was found in head and neck squamous cell carcinoma (HNSCC) with missense mutations within LZL finger and NCR domains [7]. The nucleocytoplasmic translocation of ING5 protein were observed in HNSCC [8], gastric [9], colorectal [10] and lung [11] cancers, and positively associated with their aggressive behaviors or worse prognosis. ING5 overexpression might reverse the aggressive phenotypes of gastric, breast and lung cancer cells, including proliferation, migration, invasion, epithelial to mesenchymal transition (EMT), tumor growth or metastasis [11–13]. In contrast, ING5 was found to activate β-catenin, Akt and NF-κB pathways in SGC-7901 cells, and enhance the apoptotic and chemotherapeutic resistances [12]. ING5 increased glycolysis and aerobic oxidation, which was closely linked to PFK-1 and PDPc hyperexpression in lung cancer cells [13].

In the present study, we observed the effects of ectopic ING5 overexpression on the aggressive phenotypes of breast cancer cells, and analyzed the relevant molecular mechanisms. *ING5* expression was examined in breast cancers and their precancerous diseases, and compared with the clinicopathological parameters of breast cancers to explore the roles of ING5 expression.

## RESULTS

### The effects and related molecular mechanisms of ING5 overexpression on the phenotypes of breast cancer cells

**Figure 1:**
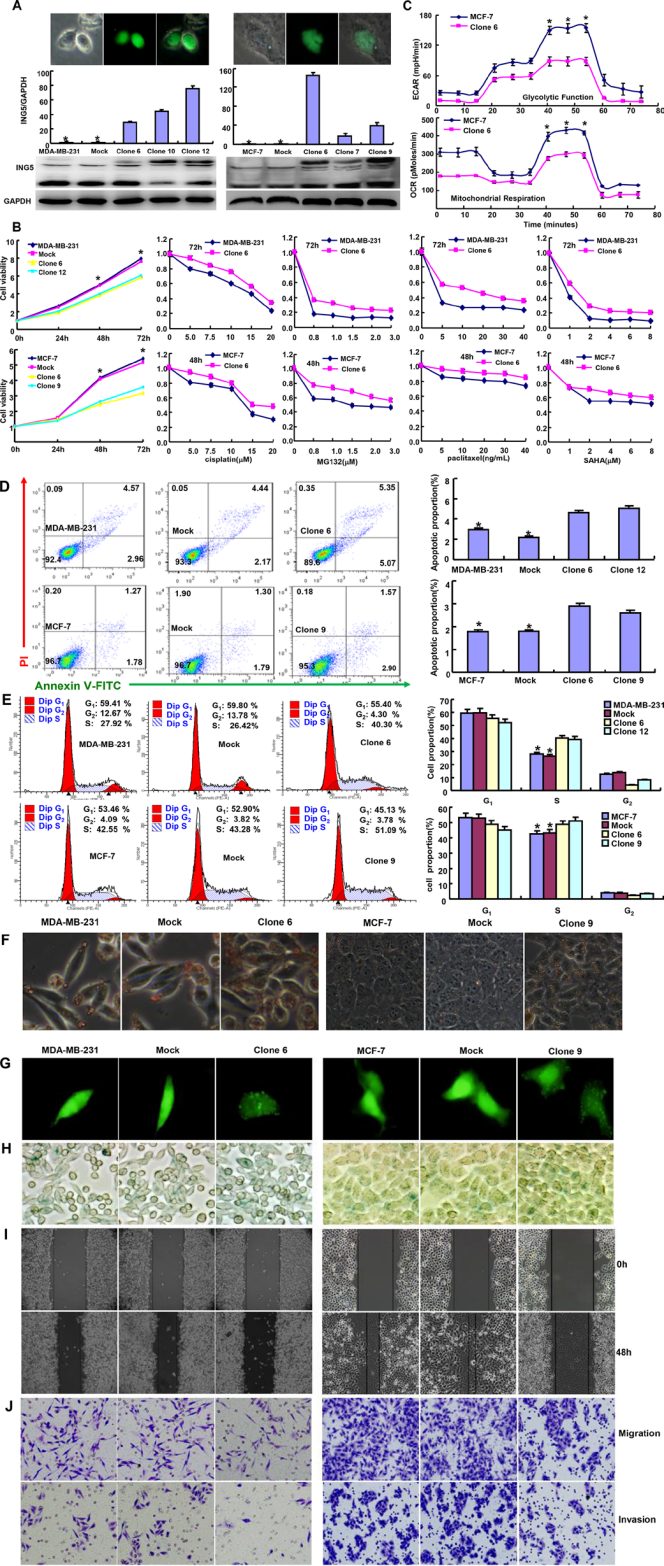
ING5 expression altered the phenotypes of breast cancer cells After transfection of pCDNA3.1-ING5, its expression became strong in MDA-MB-231 and MCF-7 cells by fluorescence, RT-PCR and Western blot (**A**). The cell viability was measured using MTT assay in both breast cancer cells and their ING5 transfectants, even treated by cisplatin, MG132, paclitaxel and SAHA (**B**). The glucose metabolism of MCF-7 and its transfectant was detected by XF-24 extracellular flux analyzers (**C**). The apoptosis, cell cycle, fat accumulation, autophagy, senescence, migration and invasion were examined by Annexin-V staining (**D**), PI staining (**E**), Oil red O staining (**F**), the transient transfection of LC-3B-expressing plasmid (**G**), β-galactosidase staining (**H**), wound healing (**I**), and transwell chamber assay (**J**) *p < 0.05, compared with the transfectants.

To clarify the roles of ING5, we successfully transfected its GFP-tagged expressing plasmid into MDA-MB-231 and MCF-7 cells, evidenced by fluorescence, RT-PCR and Western blot (Figure 1A). Compared with the control or mock, ING5 overexpression decreased cell viability and increased chemoresistance to cisplatin, MG132, paclitaxel and SAHA in both breast cancer cells (Figure 1B). It also reduced glycolysis and mitochondrial respiration, induced apoptosis and S arrest (Figure 1C–1E, *p <* 0.05). There appeared aberrant fat accumulation in ING5 transfectants, compared with the mock and control by oil red O staining (Figure 1F). According to GFP-LC-3B transfection and β-galactosidase staining, a higher level of autophagy or senescence was observed in ING5 transfectants than the control and mock (Figure 1G and 1H). Based on wound healing and transwell chamber assay, cell migration and invasion were weakened in ING5 transfectants (Figure 1I and 1J).

**Figure 2:**
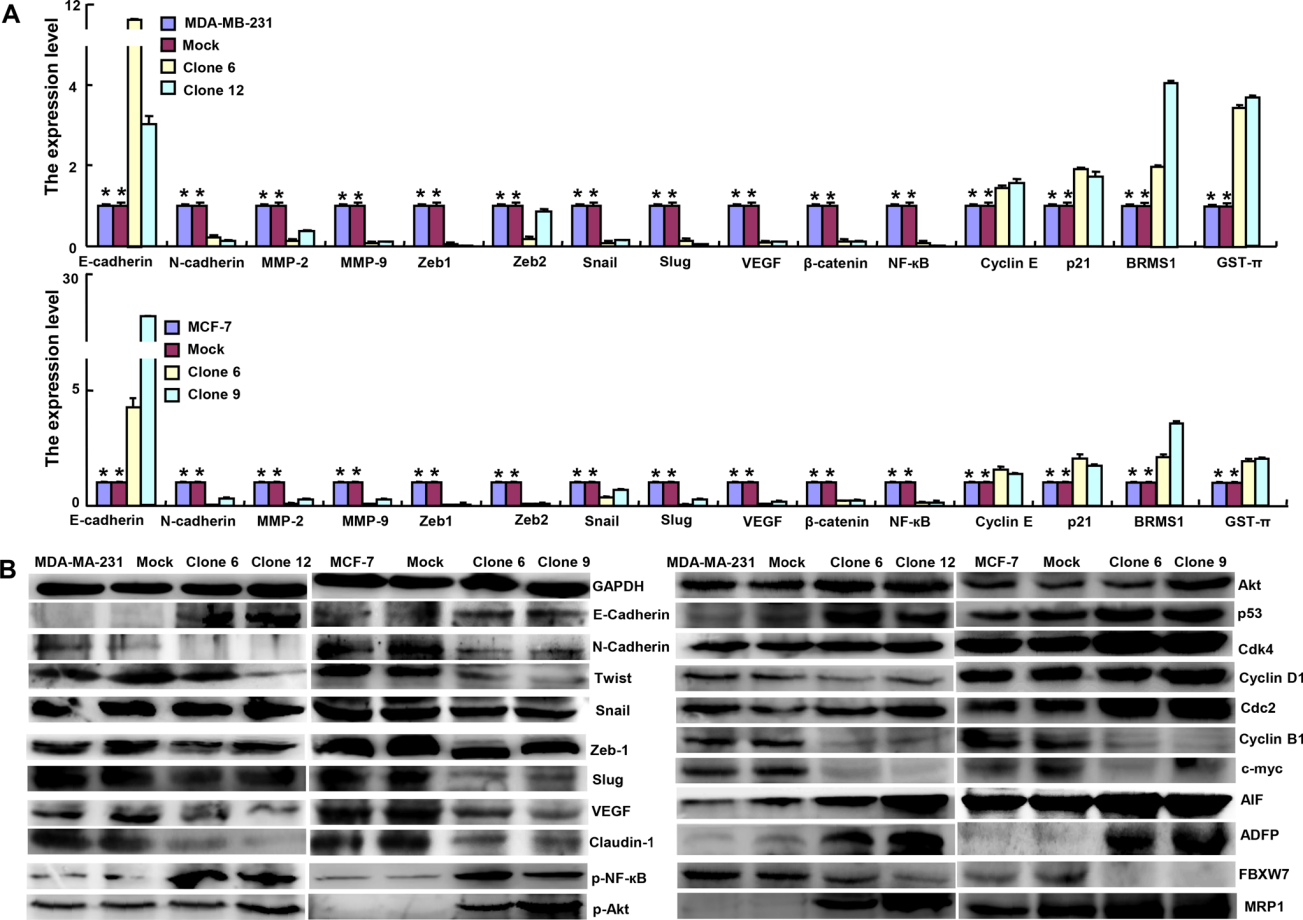
ING5 expression modulated the expression of phenotype-related molecules in breast cancer cells The phenotype-associated molecules were screened by real-time RT-PCR (**A**) and Western blot (**B**). *p < 0.05, compared with the transfectants.

At the mRNA level, ING5 overexpression increased the expression of *E-cadherin*, *Cyclin E*, *p21*, *BRMS1* and *GST-π*, but decreased the expression of *N-cadherin*, *MMP-2*, *MMP-9*, *Zeb1*, *Zeb2*, *Snail*, *Slug*, *VEGF*, β-catenin and *NF-кB* in MDA-MB-231 and MCF-7 (Figure 2A, *p <* 0.05) . According to Western blot, the expression of E-cadherin, p-NF-кB, Akt, p-Akt, p53, Cdk4, Cdc2, AIF, ADFP and MRP1 was up-regulated, but the expression of N-cadherin, Twist, snail, Zeb1, Slug, VEGF, Claudin-1, Cyclin B1, c-myc and FBXW7 was down-regulated in ING5 transfectants of both breast cancer cells (Figure 2B). However, there was no difference in Cyclin D1 expression between *ING5* transfectants and the control or mock (Figure 2B).

### The inhibitory effects of ING5 expression on the growth of breast cancer cells in nude mice

**Figure 3:**
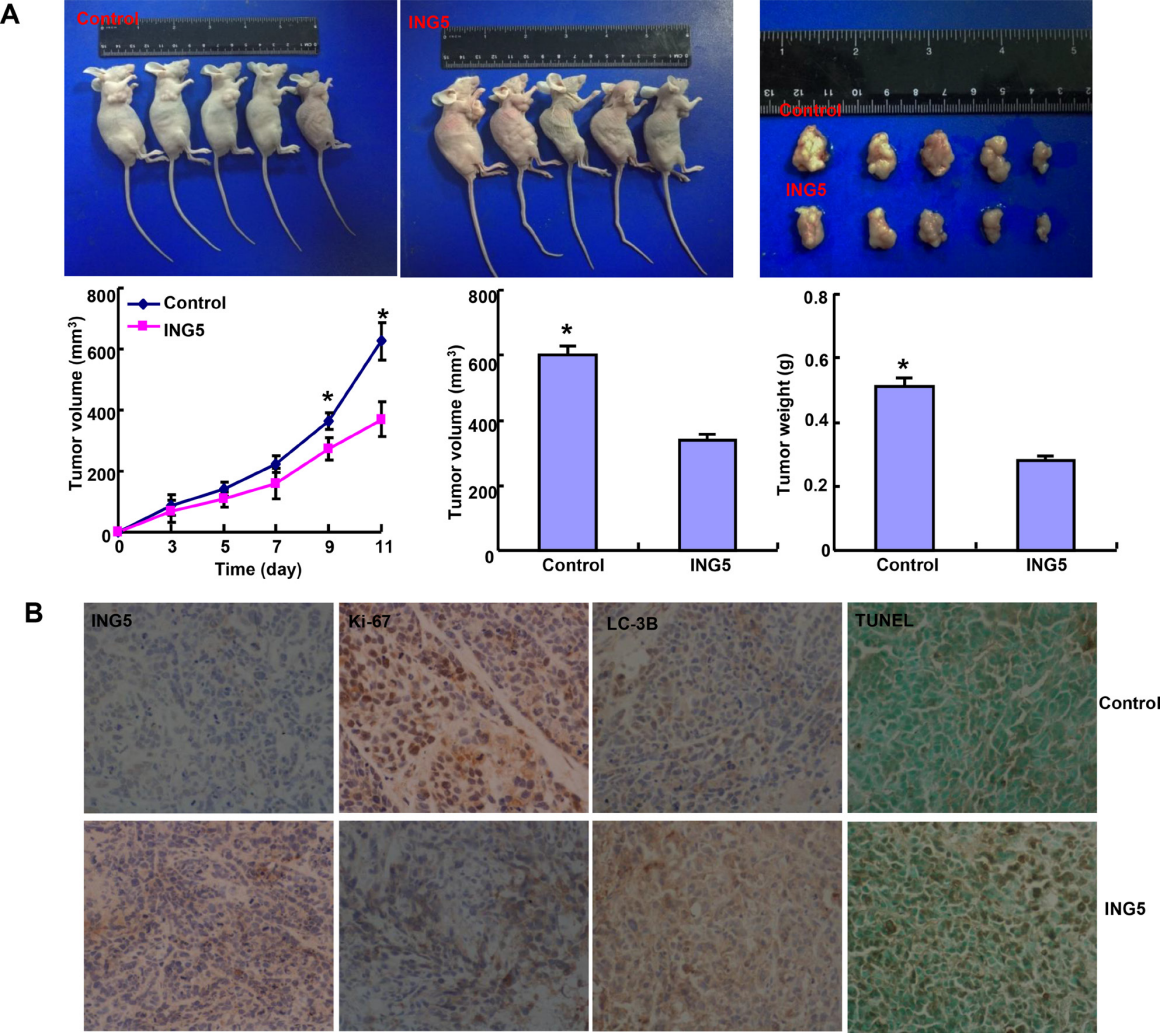
The effects of ING5 overexpression on the tumor growth of breast cancer cells in nude mice The tumor volume and weight were measured by ruling, capacity measurement and weighting (**A**) after MCF-7 and its ING5 transfectants were subcutaneously injected. Immunohistochemistry was employed for the detection of ING5 expression, ki-67 for proliferation and LC-3B for autophagy, while TUNEL for apoptotic signal (**B**). *p < 0.05, compared with the transfectants.

We subcutaneously transplanted MCF-7 cancer cells and its *ING5* transfectants into immune-deficient mice, and found that the tumor volume and weight of ING5 transfectants were smaller than the control by ruling, capacity measurement and weighting (Figure 3A, *p <* 0.05). ING5 transfectants showed lower proliferation, higher autophagy and apoptosis than the control, evidenced by ki-67 and LC-3B immunostaining, and TUNEL respectively (Figure 3B).

### The correlation of ING5 expression with the pathobiological behaviors of breast cancer

**Figure 4:**
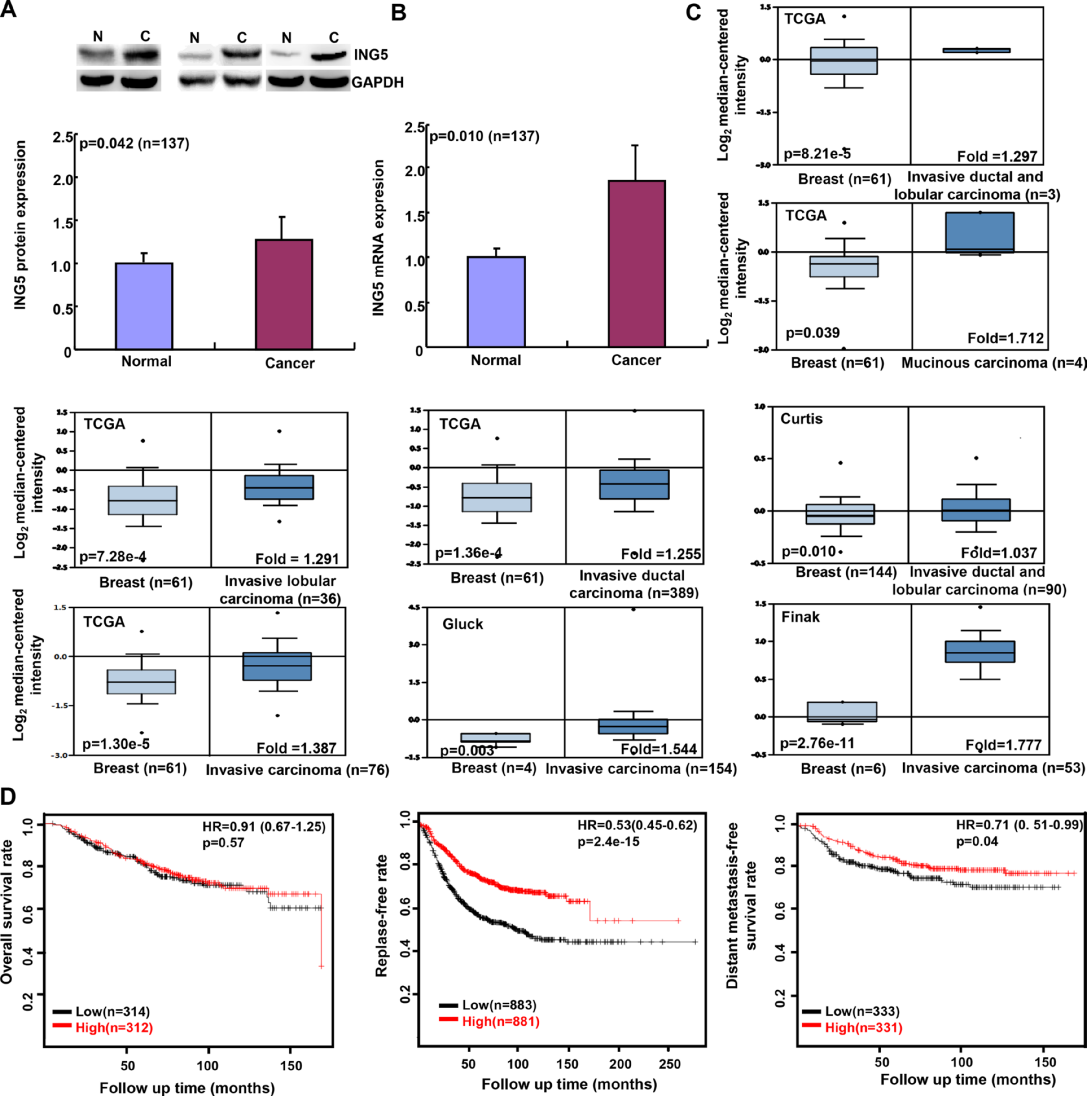
ING5 expression in breast cancer and its prognostic significance Tissue lysate was loaded and probed with anti-ING5 antibody (28kDa) with GAPDH (37kDa) as an internal control by Western blot. The densitometric analysis was performed for ING5 expression in breast cancer and matched normal tissue (**A**). *ING5* was amplified by real-time RT-PCR with *GAPDH* as an internal control (**B**). TCGA’s, Curtis’s, Gluck’s, and Finak’s datasets were employed for bioinformatics analysis to analyze *ING5* mRNA expression during breast carcinogenesis (**C**). The prognostic significance of *ING5* mRNA expression was analyzed using Kaplan-Meier plotter (**D**). Note: HR, hazard ratio.

ING5 protein level was higher in breast cancer than normal tissue by Western blot (Figure 4A, *p <* 0.05). It was the same for *ING5* mRNA according to real-time RT-PCR (Figure 4B, *p <* 0.05). In contrast, there was no association between ING5 expression and clinicopathological parameters of breast cancers at either mRNA or protein level (*p >* 0.05, data not shown). Then, we used TCGA’s, Curtis’s, Gluck’s, and Finak’s datasets to perform bioinformatics analysis, and found that *ING5* mRNA expression was higher in breast cancer than normal tissue (Figure 4C, *p <* 0.05). According to Kaplan-Meier plotter, *ING5* mRNA expression was not correlated with overall survival rate of the patients with breast cancer (*p >* 0.05), but positively with relapse- and distant metastasis-free survival rates (Figure 4D, *p <* 0.05).

**Figure 5:**
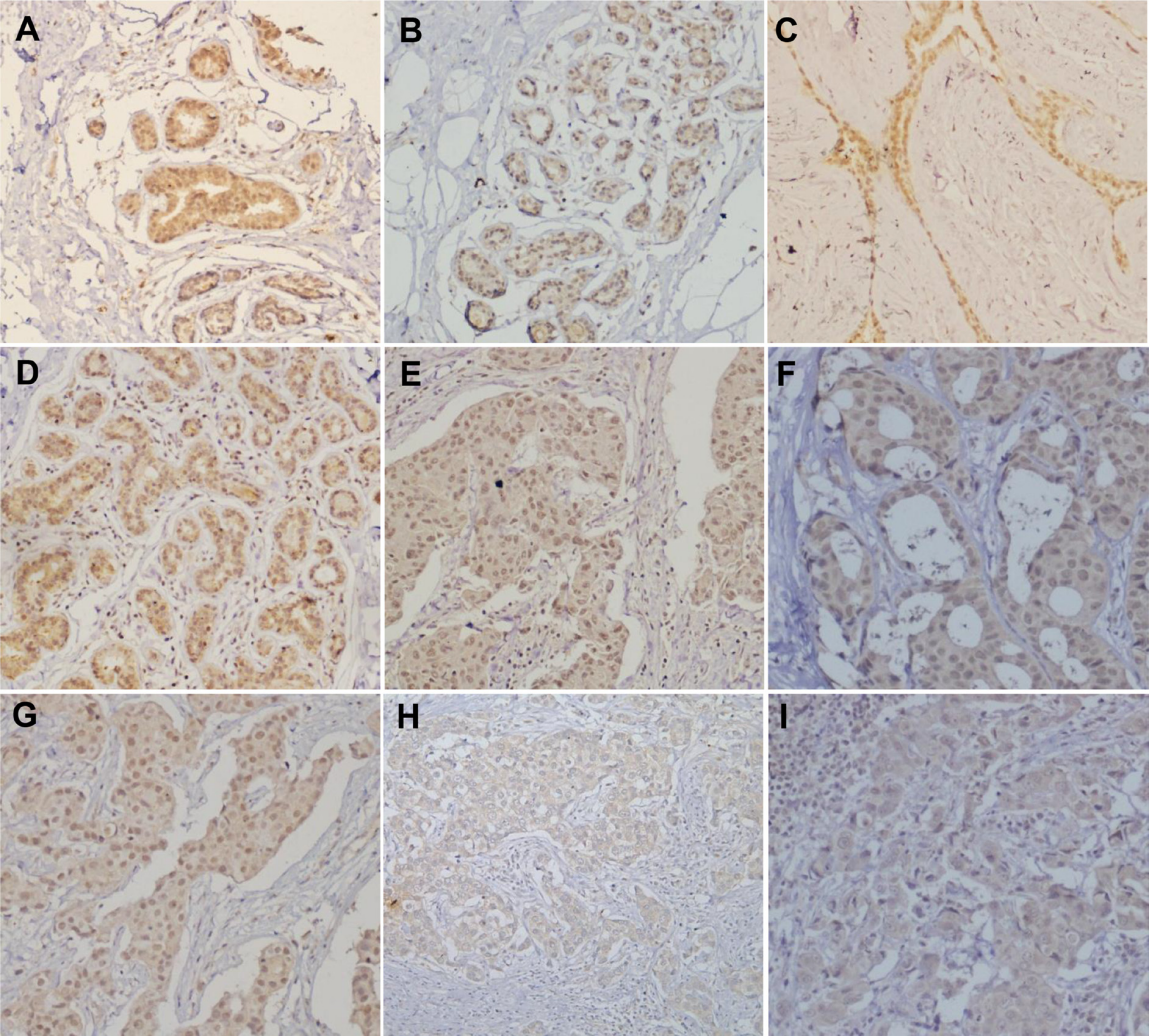
ING5 expression in breast carcinogenesis and subsequent progression ING5 protein was positively expressed in breast duct (**A**: cyt,+++; nuc,+++), acinus (**B**: cyt,+; nuc,+++), fibroadenoma (**C**: cyt,++; nuc,+++), adenomatosis (**D**: cyt,++; nuc,+++), primary cancers (**E**–**G**: cyt,++; nuc,+++; **H**:cyt,++; nuc,-) and metastatic cancer (**I**: cyt,++; nuc,+) in lymph node. Note: cyt, cytoplasmic expression pattern; nuc, nuclear expression pattern.

As shown in Figure 5 and Table 1, nuclear ING5 expression was stronger in breast normal tissue than that in fibroadenoma, adenomatosis and primary cancer (*p <* 0.05), adenomatosis than primary cancer (*p <* 0.05), and primary than metastatic cancers (*p <* 0.05, Table 1). Cytoplasmic ING5 expression was higher in fibroadenoma, adenomatosis, and primary cancer than normal tissue (*p <* 0.05, Table 1). Nuclear ING5 expression was negatively correlated with distant metastasis and p53 hypoexpression of breast cancers (*p <* 0.05, Table 2). Cytoplasmic ING5 expression was positively correlated with tumor size and ER expression of breast cancers (*p <* 0.05), and lower in triple-negative breast cancer (TNBC) than non-TNBC (*p <* 0.05, Table 2). As for non-TNBC, there was positive association between nuclear ING5 and p53 expression (*p <* 0.05), as well as between cytoplasmic ING5 expression and tumor size (Table 3, *p <* 0.05). In TNBC, nuclear ING5 expression was negatively related to distant metastasis of breast cancers (Table 4, *p <* 0.05). Stronger expression of cytoplasmic ING5 was detectable in the elder patients with breast cancer than younger ones (Table 4, *p <* 0.05). Additionally, TNBC patients showed a younger age, lager tumor size, and higher TNM staging than non-TNBC (Table 5, *p <* 0.05)

**Table 1:** ING5 expression in breast carcinogenesis and subsequent progression

Groups		Nuclear ING5 expression					Cytoplasmic ING5 expression				
	*n*	–	+	++	+++	PR(%)	–	+	++	+++	PR(%)
**Normal tissue**	91	1	4	10	76	98.9	49	29	8	5	46.2
**Fibroadenoma**	61	5	9	21	26	91.8^a^	11	28	22	0	82.0^a^
**Adenomatosis**	110	4	14	38	54	96.4^a,b^	29	56	23	2	73.6^a^
**Primary cancer**	260	24	60	101	75	90.8^a^	54	127	66	13	79.2^a^
**Metastatic cancer in lymph node**	55	11	22	16	6	80.0^b^	9	31	13	2	83.6

^a^*p* < 0.05, compared with normal tissue; ^b^*p* < 0.05, compared with primary cancer; PR, positive rate.

**Table 2:** Relationship between ING5 expression and clinicopathological features of breast cancer

Clinicopathological	*n*	Nuclear ING5 expression						Cytoplasmic ING5 expression					
features		–	+	++	+++	PR(%)	*p*	–	+	++	+++	PR(%)	*p*
**Age (years)**													
< 45	58	7	15	18	18	**87.9**	**0.648**	11	36	10	1	**81.0**	**0.165**
≥ 45	202	17	45	83	57	**91.6**		43	91	56	12	**78.7**	
**Tumor size (cm)**													
< 2.5	93	9	19	37	28	**90.3**		20	48	23	2	**78.5**	
≥ 2.5	89	6	19	36	28	**93.3**	**0.709**	11	39	29	10	**87.6**	**0.005**
**Pathological classification**													
Invasive ductal cancer	182	17	37	73	55	**90.7**	**0.657**	34	86	50	12	**81.3**	**0.189**
Invasive lobular cancer	9	1	2	4	2	**88.9**		4	3	1	1	**55.6**	
**Lymphatic vessel invasion**													
+	19	2	3	8	6	**89.5**	**0.701**	1	12	4	2	**94.7**	**0.569**
–	54	0	14	21	19	**100.0**		9	26	19	0	**83.3**	
**Lymph node metastasis**													
+	115	10	26	45	34	**91.3**	**0.812**	27	53	27	8	**76.5**	**0.977**
–	138	14	31	53	40	**89.9**		28	70	35	5	**79.7**	
**Distant metastasis**													
+	10	2	5	3	0	**80.0**	**0.026**	3	6	1	0	**70.0**	**0.605**
–	82	9	22	26	25	**89.0**		25	38	14	5	**69.5**	
**TNM staging**													
I–II	118	18	25	43	32	**84.7**		30	51	29	8	**74.6**	
III–IV	31	3	11	13	4	**90.3**	**0.284**	9	16	4	2	**71.0**	**0.330**
**ER expression**													
+	104	8	19	50	27	**92.3**	**0.293**	16	52	31	5	**84.6**	**0.044**
–	142	17	37	48	40	**88.0**		36	70	29	7	**74.6**	
**PR expression**													
+	90	6	16	45	23	**93.3**	**0.281**	14	46	26	4	**84.4**	**0.146**
–	155	18	40	53	44	**88.4**		37	76	34	8	**76.1**	
**Her2 expression**													
+	93	9	17	42	25	**90.3**	**0.586**	15	50	26	2	**83.9**	**0.452**
–	150	15	38	55	42	**90.0**		36	71	33	10	**76.0**	
p53 expression													
+	68	1	11	26	30	**98.5**	**0.035**	11	34	20	3	**83.8**	**0.887**
–	32	1	8	16	7	**96.9**		2	21	8	1	**93.8**	
**Ki-67 percentage(%)**													
< 50	138	13	23	62	40	**90.6**	**0.648**	22	68	43	5	**84.1**	**0.473**
≥ 50	23	1	5	9	8	**95.7**		3	11	6	3	**87.0**	
**Triple-negative breast cancer**													
+	92	10	28	29	25	**89.1**	**0.117**	28	44	15	5	**69.6**	**0.008**
–	168	14	32	72	50	**91.7**		26	83	51	8	**84.5**	

PR , positive rate; TNM, tumor node metastasis; ER, estrogen receptor; PR, progesterone receptor.

**Table 3:** Relationship between ING5 expression and clinicopathological features of the non-basal triple negative breast cancer

Clinicopathological Features	*n*	Nuclear ING5 expression						Cytoplasmic ING5 expression					
		–	+	++	+++	PR(%)	*p*	–	+	++	+++	PR(%)	*p*
**Age (years)**													
< 45	29	4	7	10	8	**86.2**	**0.299**	0	20	8	1	**100.0**	**0.484**
≥ 45	139	10	25	62	42	**92.8**		26	63	43	7	**81.3**	
**Tumor size(cm)**													
< 2.5	87	9	18	36	24	**89.7**		18	47	21	1	**79.3**	
≥ 2.5	74	5	14	33	22	**93.2**	**0.495**	7	33	27	7	**90.5**	**0.001**
**Pathological classification**													
Invasive ductal cancer	142	13	27	60	42	**90.2**	**0.634**	21	71	43	7	**85.2**	**0.136**
Invasive lobular cancer	9	1	2	4	2	**88.9**		4	3	1	1	**55.6**	
**Lymphatic vessel invasion**													
+	19	2	3	8	6	**89.5**	**0.701**	1	12	4	2	**94.7**	**0.569**
–	54	0	14	21	19	**100.0**		9	26	19	0	**83.3**	
**Lymph node metastasis**													
+	77	4	15	35	23	**94.8**	**0.486**	11	38	24	4	**85.7**	**0.426**
–	79	10	14	32	23	**87.3**		15	39	21	4	**81.0**	
**TNM Staging**													
I–II	51	10	9	23	9	**80.4**		9	22	15	5	**82.4**	
III–IV	7	1	0	4	2	**85.7**	**0.296**	2	2	3	0	**71.4**	**0.727**
**ER expression**													
+	104	8	19	50	27	**92.3**	**0.931**	16	52	31	5	**84.6**	**0.905**
–	49	6	9	19	15	**87.8**		7	26	14	2	**85.7**	
**PR expression**													
+	90	6	16	45	23	**93.3**	**0.743**	14	46	26	4	**84.4**	**0.800**
–	63	8	12	24	19	**87.3**		9	32	19	3	**85.7**	
**Her2 expression**													
+	93	9	17	42	25	**90.3**	**0.712**	15	50	26	2	**83.9**	**0.201**
–	58	5	10	26	17	**91.4**		8	27	18	5	**86.2**	
**p53 expression**													
+	63	1	11	25	26	**98.4**	**0.045**	10	33	18	2	**84.1**	**0.896**
–	32	2	8	15	7	**93.8**		3	20	8	1	**90.6**	
**Ki-67 percentage (%)**													
< 50	135	13	23	61	38	**90.4**	**0.771**	21	68	41	5	**84.4**	**0.354**
≥ 50	19	1	4	8	6	**94.7**		2	9	6	2	**89.5**	

PR , positive rate; TNM, tumor node metastasis; ER, estrogen receptor; PR, progesterone receptor.

**Table 4:** Relationship between ING5 expression and clinicopathological features of the triple-negative breast cancer

Clinicopathological features		Nuclear ING5 expression						Cytoplasmic ING5 expression					
	*n*	–	+	++	+++	PR(%)	*p*	–	+	++	+++	PR(%)	*p*
**Age (years)**													
< 45	29	3	8	8	10	**89.7**	**0.444**	11	16	2	0	**62.1**	**0.044**
≥ 45	63	7	20	21	15	**88.9**		17	28	13	5	**73.0**	
**Tumor size (cm)**													
< 2.5	5	0	1	1	3	**100.0**	**0.251**	2	1	1	1	**60.0**	**0.907**
≥ 2.5	10	1	4	2	3	**90.0**		2	4	1	3	**80.0**	
**Lymph node metastasis**													
+	38	6	11	10	11	**84.2**	**0.439**	16	15	3	4	**57.9**	**0.107**
–	53	4	16	19	14	**92.5**		11	29	12	1	**79.2**	
**Distant metastasis**													
+	10	2	5	3	0	**80.0**	**0.026**	3	6	1	0	**70.0**	**0.605**
–	82	9	22	26	25	**89.0**		25	38	14	5	**69.5**	
**TNM staging**													
I–II	67	8	16	20	23	**88.1**	**0.056**	21	29	14	3	**68.7**	**0.661**
III–IV	24	2	11	9	3	**91.7**		7	14	1	2	**70.8**	

PR, positive rate; TNM, tumor node metastasis.

**Table 5:** The difference in pathobiological behaviors between triple-negative and non-triple-negative breast cancers

Clinicopathological features	*n*	Triple-negative breast cancers		*p*
		+	-	
**Age (years)**				
	260	50.1 ± 10.1 (*n =* 92)	54.1 ± 10.6 (*n =* 168)	**0.004**
**Tumor size (cm)**				
	176	3.39 ± 2.08 (*n =* 15)	2.44 ± 1.05 (*n =* 161)	**0.003**
**Lymph node metastasis**				
+	132	53 (58.2)	79 (50.6)	**0.248**
–	115	38 (41.8)	77 (49.4)	
**TNM staging**				
I–II	118	67 (73.6)	51 (87.9)	**0.036**
III–IV	31	24 (26.4)	7 (12.1)	

TNM, tumor node metastasis.

## DISCUSSION

ING5 suppressed tumor growth and metastasis of lung cancer cells [11], promoted cell apoptosis and restricted proliferation of hepatocellular carcinoma cells [14], and significantly inhibited cell migration, invasion, and EMT of breast cancer cells [15]. Reportedly, ING5 had an inhibitory effect on the chemoresistance of bladder cancer and inhibited the DNA damage response pathway [16]. Here, we found that ING5 significantly suppressed proliferation, glucose metabolism, migration, invasion and tumor growth, and induced the apoptosis and autophagy, but caused the chemoresistance in breast cancer cells, in line with the previous reports [12, 13]. Therefore, ING5 may be a good molecular target for the gene therapy to reverse the aggressive phenotypes of cancer cells if its chemoresistance might be ameliorated or prevented.

In line with MET induction of ING5 in lung cancer [11], and glioma [17], ING5 overexpression was demonstrated to markedly decrease the ability of breast cancer cells to migrate and invade with E-cadherin overexpression and N-cadherin hypoexpression at both mRNA and protein levels. Zhao et al. [15] found that ING5 inhibited EMT in breast cancer by suppressing PI3K/Akt pathway. Reportedly, three families of transcription factors promoted EMT process, including Snail (Snail, Slug), Zeb (Zeb1, Zeb2) and basic Helix Loop Helix (Twist and others) [18]. Here, we for the first time investigated the molecular mechanisms about inhibitory effects of ING5 on EMT of breast cancer cells. It was found that ING5 down-regulated the expression of these transcription factors to account for its effects on EMT. Although Claudin 1 is an integral membrane protein of tight junction strands necessary for epithelial sheets [19], ING5 also reduced its expression, which should be deeply investigated in the future. Furthermore, MMP-2, MMP-9 and VEGF hypoexpression and BRMS1 hyperexpression accounted for the anti-invasion and anti-metastasis effects of ING5 because the former three promote the degradation of extracellular matrix and angiogenesis [20] and BRMS1 reduces the metastatic potential of human breast cancer and melanoma cells as a component of the mSin3a family of histone deacetylase complexes [21].

Both Cyclin D and E activate CDKs and promote G_1_-S transition, which is inhibited by p21 and p27 [22]. Cdc25B activates the cyclin dependent kinase Cdc2 for entry into mitosis [23]. p53 protein can arrest growth by holding the cell cycle at the G_1_/S regulation [24]. Therefore, ING5-mediated S arrest might be due to the combined effects of p21, Cyclin E, Cdk4, Cyclin B1 and p53. AIF transfers from the mitochondria to the nucleus, and causes nuclear DNA aggregate and fractures [25]. Its overexpression resulted in the apoptotic induction of ING5 in breast cancer cells. Adipophilin (ADFP) is a ubiquitous component of lipid droplets [26], and its overexpression enhanced the fat accumulation in ING5-overexpressing breast cancer cells.

PI3K/Akt pathway is reported to protect cancer cells against apoptotic processes [27], and NF-κB protects cancer cells from apoptosis [28]. GST-π is involved in the reductive reaction and catabolism of chemicals, MRP1 belongs to efflux transporters of the ATP-binding cassette family and pumps many foreign substances out of cells [29], and FBXW7 loss promotes chemoresistance [30, 31]. Here, ING5-associated chemoresistance might result from the activation of Akt and NF-κB pathways, MRP1 and GST-π hyperexpression, and FBXW7 hypoexpression.

In agreement with the results of gastric and colorectal cancers [9, 12], we for the first time demonstrated the higher expression level of ING5 mRNA and protein in breast cancer than normal tissues, which could also be explained by the presence of ING5 expression in stromal cells and a higher karyoplasmic ratio of cancer cells. Previously, the shift of nuclear ING5 to the cytoplasm was observed in head and neck [8], gastric [9] and colorectal [10] carcinogenesis. Cytoplasmic ING5 expression was positively linked to the aggressive behaviors and worse prognosis, but versa for nuclear counterpart [8–10], in line with our findings. Qi et al. [32] demonstrated that wild-type ING5 was localized in the nuclei of HSC-3 cells, responsible for proliferative inhibition and apoptotic induction, while two truncated fragments of aa 1-184 and aa 107-226 distributed in the cytosol for senescence. Finally, bioinformatics analysis showed that *ING5* mRNA expression was positively linked to the favorable prognosis of the cancer patients with relapse or distant metastasis free, indicating that it might be employed as a molecular marker for a good prognosis of breast cancer. Taken together, the inhibitory effects of ING5 on aggressive phenotypes of breast cancer cell might account for the positive correlation between *ING5* hypoexpression and aggressive behaviors or unfavorable prognosis of breast cancers.

The up-regulated expression and nucleocytoplasmic translocation of ING5 protein existed in breast cancer. The expression of nuclear ING5 was inversely linked to the aggressiveness of breast cancer, which might result from the ability of ING5 to suppress the proliferation, energy metabolism, migration, invasion and tumor growth, and induce apoptosis, and autophagy, senescent of breast cancer cells. ING5-associated chemoresistance might result from the dysfunction of chemoresistance-related genes, the activation of Akt and NF-κB pathways. ING5 might be employed as a potential target for gene therapy if its chemoresistant induction can be ameliorated in the future.

## MATERIALS AND METHODS

### Cell culture and transfection

MDA-MB-231 and MCF-7 breast cancer cell lines were grown in DMEM medium supplemented with 10% fetal bovine serum (FBS), 100 U/ml penicillin and 100 μg/ml streptomycin, at 37°C in 5% CO_2_. The cells were transfected with pEGFP-N1-ING5 or pEGFP-N1 vector at 24 h after seeding on dishes, or selected by G418. Cells were treated with paclitaxel (a mitotic inhibitor), cisplatin (a platinum- containing DNA crosslinker), SAHA (a HDAC inhibitor), and MG132 (proteosome inhibitor).

### Proliferation assay

Cells were seeded at 2.5×10^3^ cells per well in 96-well plates and maintained in media containing 10% FBS. At the indicated time points, 20 μl of 5 mg/ml MTT was added to each well, and then incubated for 4 h at 37°C. After that, the media was discarded, and 150 μl of DMSO was added to each well to dissolve the precipitates. The number of viable cells was counted by measuring the absorbance at 490 nm using a microplate reader (M200pro, Switzerland).

### Cell cycle analysis

1 × 10^6^ cells were collected, washed twice by PBS, and then added with 3 ml cool 70% ethanol for at least 12 h. The cells were washed twice with PBS again and incubated for 1 h at 37°C with 500 μl RNase (0.25 mg/ml). The cells were resuspended in propidium iodide (PI) at a concentration of 50 µg/ml and cycle analysis was performed by flow cytometry.

### Apoptosis assay

The apoptosis rate was determined by using PI and FITC-labeled annexin V (*KeyGEN* Biotech, Nanjing, *China*) following the manufacturer’s instructions. Flow cytometry was performed within 1 h of incubation and apoptosis was analyzed by FlowJo software.

### Oil red O staining

At aim to identify the fat accumulation, the cells were fixed in ice cold 10% formalin for 30 min and rinsed with PBS for three times. After that, the plates were incubated with pre-warmed Oil Red O solution for 15 min.

### GFP-LC-3B Assay

Twenty micrograms of GFP-LC3B was transfected into MDA-MB-231 and MCF-7 cells using Lipofectamine TM2000 in serum-free DMEM medium for 6 h, and then medium was replaced with DMEM medium + 10% FBS. The cells were incubated for an additional 24 h. Images were acquired with fluorescent microscopy.

### β-galactosidase staining

β-galactosidase staining was performed with a senescence-associated β-galactosidase staining kit (Beyotime). Cells were washed three times with PBS and fixed with 4% paraformaldehyde for 15 min. Next, the cells were incubated overnight at 37°C in dark with the working solution containing 0.05 mg/mL X-gal. Finally, cells were examined under a light inverted microscope.

### Wound healing assay

Cell migration was assessed using a wound healing assay as previously described [13].

### Transwell chamber assay

Invasion assay was performed in Boyden chambers coated with matrigel as instructed by the manufacturer (BD Biosciences) as previously reported [13]. For migration assay, the procedures were the same as above excluding membrane control insert (BD Bioscience).

### Measurement of extracellular acidification rate and oxygen consumption rate

Seahorse metabolic flux analyser was used to measure the metabolic parameters in wing discs of abx UbxFLPase. Discs were dissected and measured in bicarbonate-free Schneider medium containing 11 mM glucose and 12 mM glutamine (Sigma S9895). The rate of glycolysis can be assessed by measuring the ECAR, and the rate of respiration can be measured by monitoring the oxygen consumption rate (OCR). ECAR and OCR values were normalized to protein content in individual cells using BCA assay kit (Sigma) as previously described [13].

### Samples and pathology

The normal breast tissues (*n =* 91), fibroadenoma (*n =* 61), adeomatosis (*n =* 110), primary breast cancer (*n =* 260) and metastatic cancer in lymph node (*n =* 55) were collected from Shengjing Hospital of China Medical University. They were subjected to routine preparation of pathological blocks. The average age of the patients at surgery was 45 years (23–82 years). Among them, 115 cases are accompanied with lymph node metastasis, and 10 with distant metastasis. 137 cases of breast cancer were collected from our hospital between 2012 and 2013 and frozen in –80^°^C until protein and RNA extraction. The average age of the patients at surgery was 45 years (25–75 years). Among them, 30 cases are accompanied with lymph node metastasis. None of the patients had undergone chemotherapy, radiotherapy or adjuvant treatment prior to surgery. Informed written consent was obtained from all participants and the study was approved by Ethics Committees of both hospitals.

These sections were subjected to hematoxylin-and-eosin (HE) staining for routine pathological examination, including tumor size, lymph node metastasis and distant metastasis. The staging for each breast cancer was evaluated according to tumor-node-metastasis system and histological architecture of breast cancer was described in terms of WHO’s classification [33]. The expression of ER, PR, Her2, p53 and ki-67 was examined during clinicopathological diagnosis.

### Tissue microarray (TMA)

Representative areas of solid tumors were identified in HE-stained slices of selected tumor samples, 2mm-in-diameter tissue cores were punched out from each donor block and transferred to a recipient block using a Tissue Microarrayer (AZUMAYA KIN-1, Tokyo, Japan). Each recipient block had a maximum of 70 cores. Consecutive 4 μm-thick sections were incised from the recipient block and transferred to poly-lysine-coated glass slides.

### Xenograft models

Balb/c nude mice of 6–8 weeks were kept in a specific pathogen-free facility with a 12 h light/dark cycle. The procedures were carried out in accordance with the Animal Experiment Ethical Statement. All experimental protocols were approved by the Ethics Committee of our hospital. MCF-7 cells and their ING5 transfectants were detached by trypsinization, washed and re-suspended in serum-free medium. Subcutaneous xenografts were established by injection of 3 × 10^6^ cancer cells /mouse to the axilla (*n =* 10/group). Tumor growth was then monitored for 11 days and calculated as follows: length × width × height × 0.5. At the end of the experiment, mice from each group was anesthetized, photographed, and sacrificed for further analysis. The volume and weight of tumor were determined by capacity measurement and weighting. The tumors were subjected to routine block preparation for the following experiments.

### RNA extraction and RT-PCR

Total RNA was extracted from breast cancer cells and tissues using Trizol (Takara, Japan). The cDNA was synthesized by reverse transcription performed from 2 μg of total RNA using AMV reverse transcriptase and random primers (Takara, Japan). PCR primers were designed according to the sequences in GenBank and are listed in Supplementary Table 1. Amplification of cDNA was performed using SYBR Premix Ex Taq II kit (Takara, Japan). For relative quantification, the levels of individual gene mRNA transcripts were firstly normalized to the control GAPDH. Subsequently, the differential expression of these genes was analyzed by the 2^–ΔΔCT^ method and expressed as fold changes. The control was considered as "1".

### Western blot analysis

Cells and tissues were homogenized in RIPA lysis buffer. Protein assays were performed by Kaumas brilliant blue method. Equal amounts of protein (30μg protein each lane) were separated by 10% SDS-PAGE, transferred to a PVDF membrane using standard procedures. The membrane was blocked with 5 % skim milk in Tris-buffered saline with Tween 20 (TBST) for 1 h and incubated with primary antibody (Supplementary Table 2) overnight at 4°C. The membranes were rinsed with TBST, and incubated with anti-rabbit, anti-mouse or anti-goat secondary antibody conjugated with horseradish peroxidase (HRP, Dako, USA). Bands were visualized with LAS4010 (GE healthcare Life Science, USA) by ECL-Plus detection reagents (Santa Cruz, USA). Densitometric quantification of protein bands was performed with GAPDH as a control using Image J. The control was considered as “1”.

### Immunohistochemistry (IHC)

IHC was carried out on consecutive 4 μm-thick sections. In brief, the samples were deparaffinized with xylene three times, rehydrated with alcohol, and subjected to antigen retrieval by heating in target retrieval solution for 20 min in a microwave oven. The sections were quenched with 3% hydrogen peroxide for 5 min to block endogenous peroxidase activity. Non-specific binding was prevented by adding 5% bovine serum albumin for 5 min. The sections were incubated with rabbit anti-ING5 (Proteintech), anti-ki-67 (DAKO) or anti-LC-3B (Santa Cruz) for 2 h, and then incubated with anti-rabbit antibody conjugated to HRP (DAKO) for 1 h. After each treatment, all sections were washed three times with TBST and the binding sites were visualized with DAB. After counterstained with hematoxylin, the sections were dehydrated, cleared and mounted. Two independent observers (Ding XQ and Zheng HC) randomly selected five representative fields from each section. Any discrepancies were checked by both observers until a consensus was reached. The expression positivity was graded and counted as follows: 0 = negative; 1 = 1–50%; 2 = 50–74%; 3 ≥75%. The staining intensity score was graded as follows: 1 = weak; 2 = intermediate; and 3 = strong. The scores for ING5 positivity and staining intensity were multiplied to obtain a final score, which determines their expression as (- = 0; + = 1–2; ++ = 3–5; +++ = 6–9).

### TUNEL

Terminal deoxynucleotide transferase (TdT) mediated dUTP nick labeling (TUNEL) was performed using Apoptosis Detection Kit (Millipore, USA). According the manufacturer’s instructions, 4-μm sections were incubated with proteinase K at 37 °C for 30 min. Endogenous peroxidase activity was blocked by incubation with 3 % hydrogen peroxide in methanol. Paraffin sections were washed three times with PBS, and then subjected to TUNEL staining. The conjugated horseradish peroxidase was visualized with diaminobenzidine (DAB), followed by counterstaining with methyl green.

### Bioinformatics analysis

The individual gene expression level of *ING5* was analyzed using Oncomine (www.oncomine.org), a cancer microarray database and web-based data mining platform for a new discovery from genome-wide expression analyses. We compared the differences in *ING5* mRNA level between breast normal tissue and cancer. All data were log-transformed, median centered per array, and standard deviation normalized to one per array. The prognostic significance of *ING5* mRNA was analyzed using Kaplan-Meier plotter (http://kmplot.com).

### Statistical analysis

Results are representative of 3 different experiments, and data are expressed as mean ± standard deviation. *Spearman’s* correlation test was performed to analyze the rank data, and Mann-Whitney U to differentiate the means of different groups. *Kaplan-Meier* survival plots were generated and comparisons between survival curves were made with the log-rank statistics. SPSS 10.0 was applied to analyze all data and *p* < 0.05 was considered statistically significant.

## SUPPLEMENTARY MATERIALS TABLES


