# Supplementary material for: The nucleocytoplasmic translocation and up-regulation of ING5 protein in breast cancer: a potential target for gene therapy
Source: Oncotarget. 2017 May 17;8(47):81953–66. doi: 10.18632/oncotarget.17918 (PMC5669862; doi:10.18632/oncotarget.17918)
Supplement: Supplementary file 1 [file oncotarget-08-81953-s001.pdf]

The nucleocytoplasmic translocation and up-regulation of ING5 protein in breast cancer: a potential target for gene therapy

Supplementary Materials

Supplementary Table 1: Primers employed in the present study

Names	Primer' s sequence	Distribution	AT(oC)	Product size (bp)	Extension time (s)
E-cadherin	F:5′-CCGCCATCGCTTACA-3′<br>R:5′-GGCACCTGACCCTTGTA-3′	NM-057374.2<br>1017-1278	60	262	34
N-cadherin	F:5′-GAAAGACCCATCCACG- 3′<br>R: 5′-CCTGCTCACCACCACTA- 3′	NM-031333.1<br>2365-2581	60	217	34
MMP-2	F:5′- TGATCTTGACCAGAATACCATCGA- 3′<br>R: 5′- GGCTTGCGAGGGAAGAAGTT - 3′	XM_004057658.2<br>429-518	60	94	34
MMP-9	F:5′- TGTACCGCTATGGTTACACT - 3′<br>R: 5′- CCTCAAAGGTTTGGAAT - 3′	NM_004994.2<br>169-353	60	189	34
Zeb1	F:5′- GCTTGTGATTTGTGTGACAAGA - 3′<br>R: 5′-AATCGCATGTGTTCAATCAA - 3′	XM_017016603.1<br>6160-6305	60	146	34
Zeb2	F:5′- TCTGCGACATAAATACGA- 3′<br>R: 5′- GAGTGAAGCCTTGAGTGC- 3′	XM_019022312.1<br>3189-3296	60	108	34
Snail	F:5′-GGCTCAGTTCGTAAAGG-3′<br>R:5′-GCAGCGGTAGTCCACA-3′	NM-001032543.17-<br>363	60	357	34
Slug	F:5′-ATGCCTGTCATACCACAA-3′<br>R: 5′-GAGGAGGTGTCAGATGGA-3′	FBgn0028564<br>290-462	60	173	34
VEGF	F:5′- GCGCTCGGTGCTGGAATTTG -3′<br>R: 5′- TAGAGCAATCTCCCCAAGCCG -3′	XM_016955597.1<br>138-298	60	161	34
β-catenin	F:5′- GCTTGGAATGAGACTGCTGA -3′<br>R: 5′- CTGGCCATATCCACCAGAGT -3′	X87838<br>2221-2334	60	114	34
NF-kB	F:5′- GTGGACTACCTGGTGCCTCTA -3′<br>R: 5′- GTCCTTGGGTCCAGCAGTT -3′	XM_011532006.2<br>437-630	60	194	34
Cyclin E	F: 5′-GGATGTTGACTGCCTTGA-3′<br>R: 5′-CGCACCACTGATACCCT-3′	BA000005<br>1044-1150	60	107	34
p21	F: 5′-ACTGTCTTGTACCCTTGTGCC-3′<br>R: 5′-AAATCTGTCATGCTGGTCTGC-3′	XM_003950827<br>572-679	60	108	34
BRMS1	F: 5′- CTGTCCAGCCTCCAAGCAAAG-3′<br>R: 5′- TCTAGGTCCAGCATCTCACTGAC-3′	XM_001171301.4<br>278-476	60	199	34
GST-π	F: 5′- CGGGCAAGGATGACTATGTGA -3′<br>R: 5′- GGGCTAGGACCTCATGGATCA -3′	BC044846.1<br>1137-1475	60	339	34
ING5	F: 5′- CAAGGAATACAGTGACGACAA -3′<br>R: 5′- AAAATCACTGCCCTCCATC -3′	NM_001330162.1<br>272-407	60	136	34
GAPDH	F: 5′-CAATGACCCCTTCATTGACC-3′<br>R: 5′- TGGAAGATGGTGATGGGATT-3′	NM_ 002046.3<br>201-335	60	135	34

AT = annealing temperature.

Supplementary Table 2: The antibodies used in the present study

Names	Source	Company
GAPDH	Rabbit	Wanleibio
ING5	Rabbit	Proteintech Biotech. Inc.
E-Cadherin	Rabbit	Abcam
N-Cadherin	Mouse	Abcam
Twist	Rabbit	Wanleibio
Snail	Rabbit	Wanleibio
Zeb1	Rabbit	Wanleibio
Slug	Rabbit	Abcam
VEGF	Rabbit	Santa Cruz Biotech. Inc.
Claudin-1	Mouse	Santa Cruz Biotech. Inc.
p-NF-κB	Rabbit	Santa Cruz Biotech. Inc.
p-Akt	Rabbit	Santa Cruz Biotech. Inc.
Akt	Rabbit	Santa Cruz Biotech. Inc.
p53	Rabbit	Wanleibio
Cdk4 (C-22)	Rabbit	Santa Cruz Biotech. Inc.
Cyclin D1 (H-295)	Rabbit	Santa Cruz Biotech. Inc.
Cdc2 (B-6)	Mouse	Santa Cruz Biotech. Inc.
Cyclin B1 (GNS1)	Mouse	Santa Cruz Biotech. Inc.
c-myc (9E10)	Mouse	Santa Cruz Biotech. Inc.
AIF(E-1)	Mouse	Santa Cruz Biotech. Inc.
ADFP	Rabbit	Abcam
FBXW7	Rabbit	Wanleibio
MRP	Mouse	Abcam
